# COVID-19 and mental health: Building back better or reimagining a new way forward?

**DOI:** 10.1371/journal.pmed.1004216

**Published:** 2023-04-06

**Authors:** Mark Tomlinson, Marguerite Marlow

**Affiliations:** 1 Institute for Life Course Health Research, Department of Global Health, Stellenbosch University, Cape Town, South Africa; 2 School of Nursing and Midwifery, Queens University, Belfast, United Kingdom

## Abstract

In this Perspective, Mark Tomlinson and Marguerite Marlow argue that equitable improvements in mental health outcomes cannot be achieved without first dismantling colonial and paternalistic approaches to global mental health.

In the early months of the Coronavirus Disease 2019 (COVID-19) pandemic, data were mixed on the pandemic’s impact on mental health. Globally, suicide did not increase as was initially feared, and in some countries, there was evidence of a decline in suicide rates [1]. In a number of longitudinal population-based studies in high-income countries (HICs) comparing the prevalence of current mental disorders before and during the pandemic [2–4], only 1 reported an increase in mental disorders [2]. In contrast, the World Health Organization reported a significant increase in mental health problems in the first year of the pandemic, with women more affected than men and younger people more affected than older adults [5]. Although research from low- and middle-income countries (LMICs) was limited, available data showed that these settings were also affected [5]. Any potential doubts about the impact of COVID-19 on mental health have been largely put to rest by the papers in this special issue.

There is, however, a nagging sense that we have been here before. How would we imagine that 3 years of fear, millions of deaths, misinformation, school closures, and economic recession would not have a massive impact on mental health? How would we expect these societal conditions to not drastically determine people’s wellbeing in a way that continued to evolve in tandem with the pandemic [6,7]? That mental health is socially determined is not novel [8]. It should also come as no surprise that the pandemic has affected mental health in LMICs in different ways than in HICs [9]. In the same way that LMICs experienced inequitable access to well-known forms of protection against COVID-19 (masks, physical distancing, and vaccines), so too did they lack equitable access to the protective factors important for mental resilience and support during the pandemic: housing and food security, jobs with flexibility, adequately resourced health services, opportunities for learning, and access to social support and protection [10]. The extreme inequities were bound to lead to inequities in outcome/mental health challenges.

As important as the papers in this special issue are in highlighting the impact of the pandemic on mental health, perhaps their greatest value is in showing how little of what is known has been acted on. Epidemiological studies often run the risk of “admiring the problem,” of merely describing the social determining of health, while inadvertently allowing the gravity of the problem to substitute for real action. Breilh uses the phrase “social determining,” rather than “social determinants,” arguing that traditional “social determinants” focus on the discrete factors that affect health and wellbeing (such as education or housing—the “causes of the causes”), while social determining brings the structural factors at population level that lead to inequities and the interrelationships among them to the forefront [11]. Considering what is already known about the importance of the social determining of mental health and how these conditions affect millions of people in LMICs, how did all countries fail so spectacularly at addressing these key determinants to “survival” as part of the pandemic response? What can be learned in terms of addressing longstanding challenges related to mental health in the face of future global emergencies?

Of course, the answers to these questions are numerous. In this Perspective, we focus on the type of knowledge that is currently prioritised, and how lip service is paid to the social determining of mental ill-health. We believe that the root causes of this problem are to some extent located in often well-meaning “global health” efforts that seek to maintain—however unconsciously—historic power structures in governance and society. These power structures are held in place by an enduring colonial pattern of knowledge and solutions flowing from north to south [12], from paternalistic adults to children and adolescents [13], and the attitude of Western science toward indigenous knowledge systems [14]. A radical rethink of the dominant scientific traditions that underpin current approaches to epidemiology and “global” health—including global mental health—is required. The current approach focuses narrowly on the distribution of disease or health-related states, accompanied by an often oversimplified and narrow description of causes and risk factors, a crude dichotomising of the complexity of adversity [15]. The focus is on counting “adversities” [16], rather than on the complex pathways, mechanisms, and interplay of structural factors and biology through which adversities affect people’s lives.

In this context, it is questionable whether the oft repeated post COVID-19 term “building back better” goes far enough. Returning to “business as usual” will not serve to achieve sustained long-term recovery, especially not for those who were and continue to be disproportionately affected. It will also not be enough to foster the resilience and preparedness required to tackle current climate breakdown and (inevitable) future global emergencies. Is it possible that some dismantling has to take place first in order to build back better? Alternative approaches that have their foundations in connection and healing, rather than extraction and exploitation, are essential [17]. A relevant initiative in this regard is the One Health Approach that aims for a unified approach to balance the health of the environment, the health of people, and that of animals (https://www.onehealthcommission.org/). Key to this approach is developing solutions that do not simply treat symptoms but address root causes and their impacts, as well as advocating for transdisciplinary and community-oriented cooperation [18].

A stark lesson from the COVID-19 pandemic was the realisation that a radical rethink about how we engage with children and adolescents to address mental health and wellbeing is needed. While older populations were among the most affected by COVID-19 in terms of illness severity and mortality, the pandemic has greatly affected the wellbeing of children and adolescents [19]. Admirable progress has been made with “nothing about me without me” as a principle of engaging with people with mental disorders [20]. But the same cannot be said with regard to a similar commitment when engaging with children and adolescents. To the best of our knowledge, not a single government advisory group for the pandemic included representation from young people. Children and adolescents were not consulted about school closures, loss of their peer groups at particularly sensitive developmental periods, and were expected to make significant sacrifices to protect others from a virus that was significantly less dangerous for them. Of course, they may have been prepared to close schools, but no one asked.

The extent to which young people are routinely excluded from decisions about their own lives has long been a subject of research and enquiry. In the early 1980s, Lofquist described the prevailing attitude towards youth engagement as one in which youth were treated as objects rather than as partners [21]. The UN Convention of the Rights of the Child in an attempt to correct this, enshrined the rights of children and adolescents to participate in all matters related to their lives in 1989. It could be argued however, that the translation of this into actual change in the lives of marginalised children has been minimal. More recently, the terms “adultism” and “ageism” have been used to describe the lack of respect for the needs, competence, and potential of young people [22], resulting in adults believing that they have the right to make decisions for young people without their consent [23]. The most cursory analysis of current educational institutions and legal systems reveal the extent to which they are redolent with “adultism.” Pandemic responses across the world have mirrored this norm, allowing no actual seat for children and adolescents at the decision-making table.

The recent WHO-UNICEF-Lancet Commission puts forward a strong case for investing in, and prioritising, the world’s children [24]. This should include empowering children and adolescents to become decision makers in their own lives and society at large. This will require a deep form of youth engagement rather than the current “tick-box” approach. A transformation in how children are seen and treated is required, a move away from a paternalistic attitude of “we [as adults] know best.” Particular attention would need to be paid to governance issues and challenges that act as adult-made barriers to engaging with young people. It would also mean advocating for investments to address social norms that ignore children’s voices and creating policies that promote the full and effective participation of young people [13].

The aftershocks of the pandemic will continue to ripple across societies and its full impact on mental health and wellbeing may take years to become fully apparent. The findings in this special issue have important implications for future research and global mental health measures for responding to global emergencies. Epidemiology, treatment, prevention, and promotion are key aspects of our response to the increasing burden of mental ill health. However, we cannot continue to pay lip service to the social determining of health [11] and use the concept as little more than an additional set of risk factors to measure and count. Building back better will require a people-centered recovery to achieve wellbeing “for all,” with a central focus on inclusiveness and reducing inequality. Equitable improvements in mental health outcomes will not happen without a fundamental restructuring of a grossly inequitable world, as well as children and adolescents being afforded real participation and power in global mental health discussions and solutions.
